# ﻿*Primulalongipes* subsp. *bayburtensis* Sefali, Yapar & Demir (Primulaceae): A new taxon from north-eastern Anatolia, Türkiye

**DOI:** 10.3897/phytokeys.256.150268

**Published:** 2025-05-23

**Authors:** Abdurrahman Sefalı, Yakup Yapar, İbrahim Demir, Bayram Yurtvermez, Ali Murat Keser

**Affiliations:** 1 Department of Primary Education, Faculty of Education, Bayburt University, Bayburt, Turkiye; 2 Department of Molecular Biology and Genetics, Faculty of Arts and Sciences, Bingöl University, 12000 Bingöl, Turkiye; 3 Department of Biology, Faculty of Arts and Sciences, Bitlis Eren University, Bitlis, Turkiye; 4 Department of Organic Farming Management, Faculty of Applied Sciences, Bayburt University, Bayburt, Turkiye; 5 Department of Plant and Animal Production, Yuksekova Vocational School, Hakkari, Turkiye

**Keywords:** *Crystallophlomis*, Flora of Türkiye, new taxon, *Primula*

## Abstract

The new taxon, Primulalongipessubsp.bayburtensis Sefali, Yapar & Demir, **subsp. nov.** (Primulaceae) is described and illustrated from Bayburt Province, Türkiye. It is morphologically assigned to Primulasect.Crystallophlomis based on its long scapes, lanceolate and denticulate leaf with long petiole and hairless habit, fruits longer than calyx and covered by farina on the scape. This new taxon is similar to *Primulalongipes* and P.nivalissubsp.turkestanica; it can also be easily distinguished by its calyx divided ratio, thurm flowers (unwidened corolla tube shape and length) and capsules (length) futures.

## ﻿Introduction

The genus *Primula* L. (1753:142) is found in temperate parts of the Northern Hemisphere, extending from East Asia and Europe to the subarctic zone. Based on morphological features, the ca. 500 species in the genus have been divided into 37 sections (Richards 2003; Smith and Fletcher 1942). With about 300 species spread throughout 24 sections, the Himalayan-Hengduan Mountains in particular, located in south-western China, are a hotspot for *Primula* diversity (Hu 1994; Hu and Kelso 1996).

Since the majority of *Primula* species produce lovely, eye-catching flowers, several species that are very ornamental and can be easily cultivated and multiplied are popular ornamental plants. As pot and garden plants, *P.malacoides*, *P.obconica* and P.×polyantha, for instance, are currently considered key floricultural crops (Kato et al. 2018). These attractive flowering plants of *Primula* have attracted the attention of not only gardeners, but also researchers. *Primula* flowers have been popular for 150 years (Wu et. al. 2023) since Darwin made his pioneering work on flower polymorphism in *Primula* (Darwin 1877). The genus species are mostly (92%) distyles (Wang et al. 2021) that, usually, consist of two flower morphs (i.e. long-styled or short-styled) that differ reciprocally in stigma and anther height in a tubular flower (Barrett 2019).

Primulasect.Crystallophlomis (also referred to as Nivalid *primulas*) are often long, erect (about 40 cm), with a basal rosette of lanceolate, somewhat fleshy leaves and typically have white or pale yellow farinose below. The section has the potential to develop multiple flower whorls and pink, purple, white, cream or generally magenta flowers. Due to its usual location, its buds are covered by snow for a considerable amount of time. *Primulanivalis* Pallas, which is found in central Asia and southern Siberia, is the section’s type species. It has a robust habit, broad, lanceolate leaves with serrate margins, a tall, farinose scape up to 40 cm high and distyles flowers (Kelso 1987). P.sect.Crystallophlomis has four subsections: subsection Agleniana, subsection Calliantha, subsection Crystallophlomis and subsection Maximowiczii (Rankin 2012). Subsection Crystallophlomis has rotational symmetrical flowers, ca. 2.5 cm resting buds, farinose, green or white petioles, corolla lobes not reflexed and broad, and capsules dehiscing with valves (Rankin 2012).

Türkiye has 12 taxa of *Primula* belonging to eight species that were recognised by Lamond (1978). In the last decade, a hybrid, *Primula×uzungolensis*, was identified in north-eastern Türkiye (Terzioğlu et al. 2012) in the Soğanlı and Kaçkar Mountains at Trabzon, Gümüşhane, Rize, Artvin Province. The north-eastern part of Türkiye contains the richest species of the *Primula* and *Androsace* taxa. *Primula* is represented by 12 taxa and *Androsace* is represented by 8 taxa. (Lamond 1978; Terzioğlu et al. 2012; Sefalı 2021; Sefalı and Yapar 2022).

**Figure 1.**
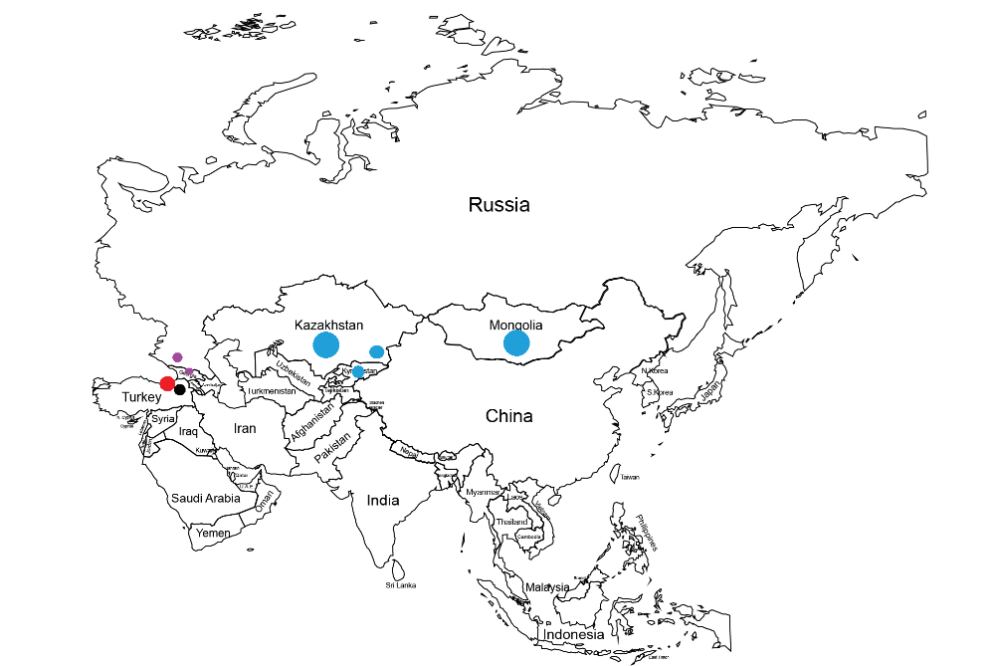
Distribution map for Primulalongipessubsp.bayburtensis (red circle), P.longipessubsp.longipes (black circle), P.nivalissubsp.turkestanica (blue circle) and *P.crassifolia* (purple circle).

Our field study was undertaken in Bayburt Province within the north-eastern area of Türkiye. This Province belongs to the Soğanlı Mountains (Kırklar Mountain) chain. As a result of the field work carried out three times in June and July 2024, the new taxon was found in the south valley of Kırklar Mountain (Fig. 1). The authors noticed an interesting Nivalid *Primula* that resembled *Primulalongipes* Freyn & Sint. (1896: 141), but this had a distinct long corolla tube, longer capsule and linear bracts. As a result, it was decided that this plant could be a new taxon and was named as Primulalongipessubsp.bayburtensis.

## ﻿Material and methods

The new taxon, Primulalongipessubsp.bayburtensis, was compared with herbarium specimens at EGE, ISTE, BIN, VANF, KNYA, ANK and AIBU. Additionally, some digital herbarium materials were examined (E, K, BR, WU herbaria and G) (acronyms according to Thiers (2025)). In addition, the relevant literature was reviewed (Fedorov 1952; Lamond 1978; Kelso 1987; Hu 1994; Hu and Kelso 1996; Richards 2002; Gültepe et al. 2010; Terzioğlu et al. 2012; Rankin 2012; Kato et al. 2018). Using a stereo-binocular microscope equipped with millimetric rules, extensive morphological measurements were carried out on the new taxon material and compared with its closely related species, *Primulalongipes*. Since the new taxon was found in only one valley, 20 individuals were included in the study.

A seed micrograph was taken with a scanning electron microscope (SEM). Seed properties were interpreted according to Morozowska et al. (2011). Palynological investigations were conducted with both light and scanning electron microscope (SEM). Pollen slides were prepared using the Wodehouse technique (1935) for light microscope studies. The averages and standard deviations of the measurements were calculated by light microscope. Measurements were taken for at least 25 pollen grains. The pollen terminology follows Faegri and Iversen (1975) and Punt et al. (2007).

## ﻿Results and discussion

### ﻿Taxonomic treatment

#### ﻿ Primula longipes Freyn & Sint. subsp. bayburtensis

Taxon classificationPlantaeEricalesPrimulaceae

Sefali, Yapar & Demir subsp. nov.

C5BA900F-E0BB-5DC4-BA07-4E106D598FA2

urn:lsid:ipni.org:names:77362178-1

Fig. 4

##### Diagnosis.

Primulalongipessubsp.bayburtensis differs from P.longipessubsp.longipes in its longer corolla tube and thurm flowers unwidening corolla throat. While Primulalongipessubsp.bayburtensis’s corolla tube ca. 14 mm and calyx/corolla tube length ratio is 0.5, P.longipessubsp.longipes’s corolla tube ca. 12 mm and calyx/corolla tube length ratio is 0.7 (Fig. 3).

##### Type.

Türkiye. Bayburt • Kırklar Mountain, southern valley, river side, stony and wet places, 2950 m elev., 15 June 2024, *A. Sefali, Y. Yapar & İ. Demir 1052* (holotype: VANF!; isotypes: BIN!).

##### Etymology.

Primulalongipessubsp.bayburtensis is named after the geographical province. This epithet is *bayburtensis* (in Turkish, Bayburt), refering to Bayburt Province, north-eastern Türkiye. The Turkish name for this taxon was chosen as “Bayburt çulhası” (Menemen et al. 2016).

##### Description.

Sturdy perennial, 15–50 cm, basal bud scales long stocks formed by overlapping petioles. ***Roots*** thickish and fibrous. ***Stem*** shortly puberulent, farinose. ***Leaves*** rosette, petiole broadly winged, 6–8 × ca. 1 cm. ***Leaf lamina*** elliptic-lanceolate 6–22 × 1.5–3 cm and near petiole shortly puberulent, farinose, base gradually thinning. Leaf margin irregularly blunt denticulate and apex bluntly acute. ***Scapes*** 10–38 cm, elongated to 45 cm in fruit. Inflorescence, 4–30-flowered (sometimes 2-whorled) at umbels. ***Bracts*** linear lanceolate, generally filiform, 2–13 mm. Flowers heterostylous. ***Pedicel*** 8–12 mm, elongated to 2–5.5 cm in fruit. ***Calyx tubular***, 5–7 mm, divided to 3/5. ***Calyx teeth***, 3.5–4.5 mm, lobes linear to lanceolate. ***Corolla tube*** ca. 14 mm, lobes 6–10 mm, ± broadly oblong, entire, magenta. ***Pin flowers***, stamens 4–5 mm above base of corolla tube, style longer than calyx. ***Thrum flowers***, stamens equalling or above (1–1.5 mm) apex of calyx, style ca. 4 mm. ***Capsule*** cylindrical, 2–4.5× as long as calyx. ***Seed*** elliptic to deltoid, yellowish, 2 × 1.5 mm (Fig. 4).

##### Phenology.

Flowering from June to July; fruiting in August.

##### Seed and pollen morphology.

(Fig. 5) Seed grains of P.longipessubsp.bayburtensis are eliptic to deltoid, yellowish, 2 × 1.5 mm, tuberculate, P.longipessubsp.longipes is elliptic, brownish, 2 × 1.5 mm, tuberculate. Pollen grains of P.longipessubsp.bayburtensis are prolate-spheroidal (P/E = 1.04 μm), polar axis (P) 19.17 μm (max. 20.98 μm, min. 17.73 μm), equatorial axis (E) 18.19 μm (max. 19.44 μm, min. 16.75 μm), radially symmetrical, isopolar, triparasyncolpate. Shape in polar view triangular. Exine 0.91 μm (max. 1.15 μm, min. 0.65 μm), intine 0.45 μm (max. 0.55 μm, min. 0.33 μm). Ornamentation of exine microreticulate. Additionally, pollen grains of P.longipessubsp.longipes are prolate-spheroidal (P/E = 1.07 μm). Polar axis 20.22 μm (max. 21.54 μm, min. 18.79 μm), equatorial axis 18.75 μm (max. 20.14 μm, min. 17.14 μm) radially symmetrical, isopolar, triparasyncolpate. Shape in polar view triangular. Exine 0.96 μm (max. 1.25 μm, min. 0.72 μm), intine 0.51 μm (max. 0.58 μm, min. 0.41 μm). Ornamentation of exine microreticulate.

##### Distribution and ecology.

**Figure 2.**
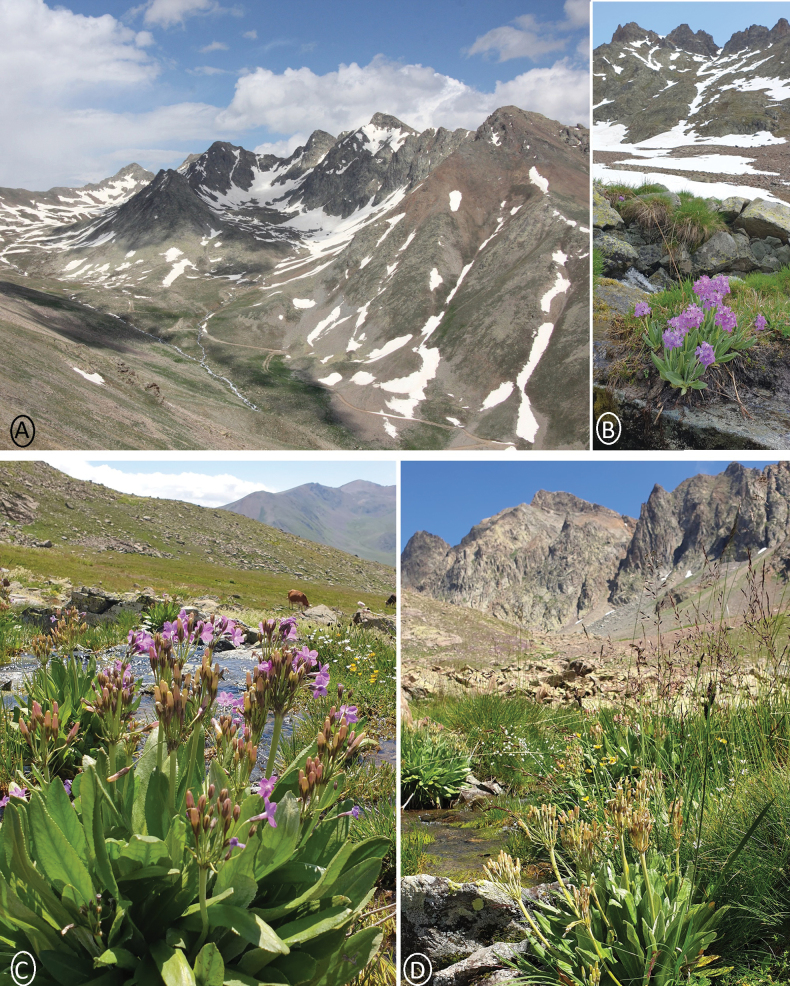
Kırklar Mountain: **A** southern valley of Kırklar Mountain **B** habitat of Primulalongipessubsp.bayburtensis (early period) **C** late period of new taxon **D** fruiting time of new taxon.

(Fig. 2) The new taxon is a local endemic restricted to the south valley of Kırklar Mountain in Bayburt Province, North-eastern Türkiye. It is an Euxine element (Figs 1, 2). The habitat of the Primulalongipessubsp.bayburtensis is stream banks and it grows in open alpine areas. The new subspecies grows in the near vicinity of *Drabanemorosa* L., *Cardamineraphanifolia* Pourr., *Primulaalgida* Adams, *P.auriculata* Lam., P.elatiorsubsp.amoena (M.Bieb.) Greuter & Burdet, *Pedicularisnordmanniana* Bunge, *Ericaspiculifolia* Salisb., *Scillaalinihatiana* Aslan & Yıldırım, *Silenelazica* Boiss. (it is found near stony places), *Erodiumhendrikii* Alpınar, *Pinguiculabalcanica* Casper, *Heracleumpastinacifolium* K.Koch, *Saxifragaexarata* Vill., *Minuartiaaizoides* (Boiss.) Bornm. and *Poa* sp.

**Figure 3.**
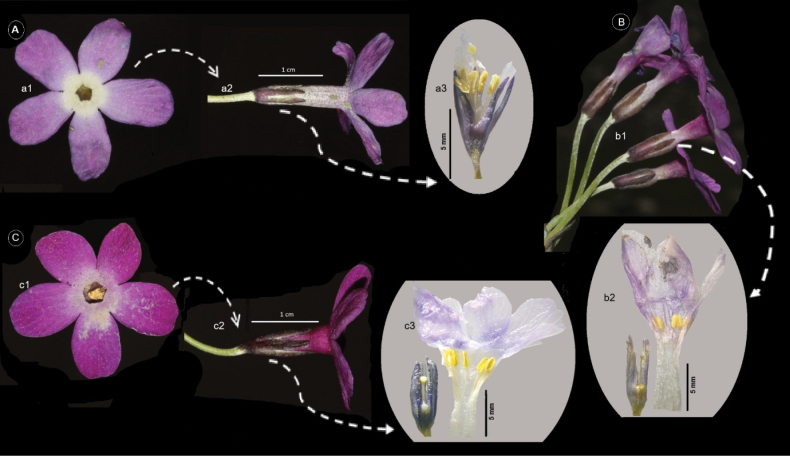
Morphologically related taxon in terms of flower, corolla tube and calyx ratio. **A** Pin flower of Primulalongipessubsp.bayburtensis**B** thrum flowers of P.longipessubsp.bayburtensis**C** thrum flowers of P.longipessubsp.longipes.

**Figure 4.**
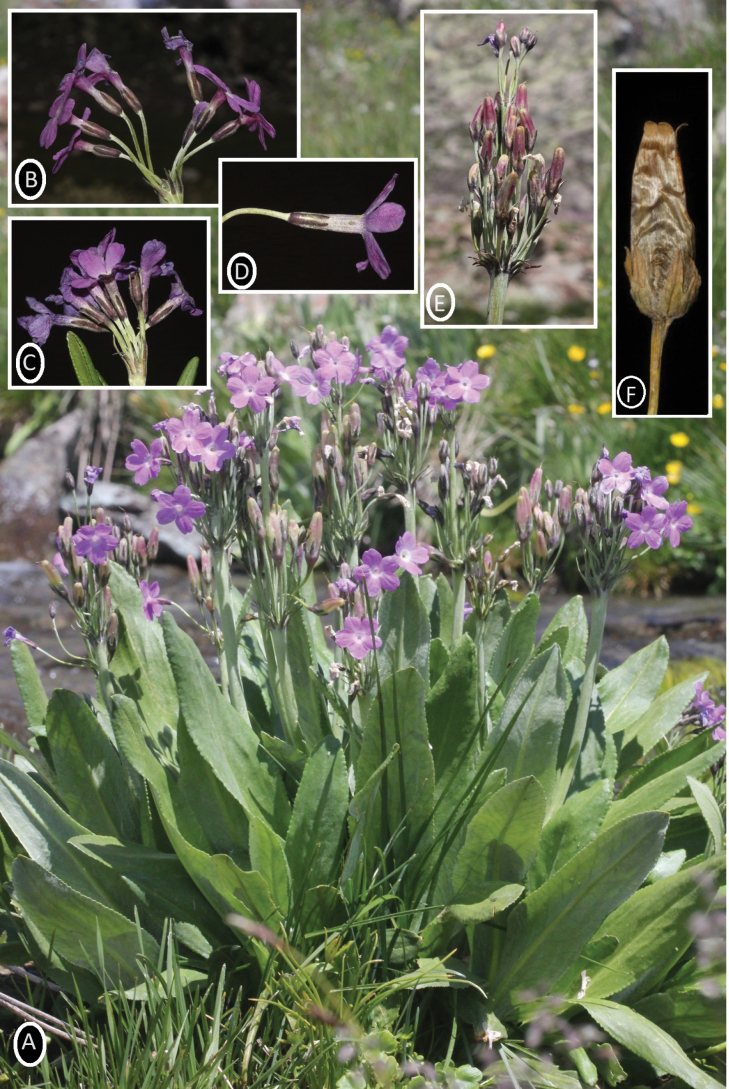
Primulalongipessubsp.bayburtensis: **A** Habitus **B**, **C** inflorescence **D** flower (calyx and corolla tube) **E** fruits and bracts **F** fruit.

**Figure 5.**
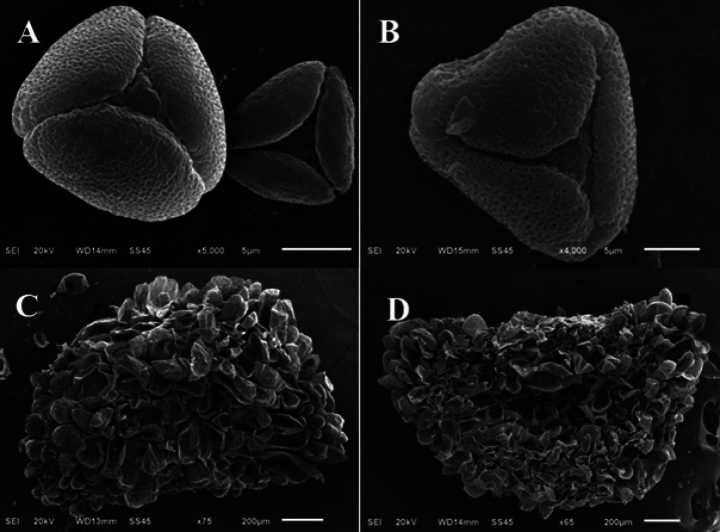
SEM pollen (thurm flowers) and seed grain photographs: **A–C**Primulalongipessubsp.bayburtensis**B–D**P.longipessubsp.longipes.

##### Taxonomic relationships.

Primulasect.Crystallophlomis members are generally distributed in highly mountainous places. The new taxon belongs to the Primulasect.Crystallophlomis and subsection Crystallophlomis (Rankin 2012). Primulalongipessubsp.bayburtensis is closely related to P.longipessubsp.longipes, P.nivalisPall.subsp.turkestanica (Haage & Schmidt) Kovt. and *P.crassifolia* Lehm. as they all belong to the same section (section Crystallophlomis). According to Lamond (1978), in Turkish Flora, *P.longipes* is one of the most beautiful of the Nivalid *Primulas* and a western vicarial of the Caucasian white-flowered *P.bayernii*, which is a synonym of *P.crassifolia* now, and also P.nivalisPallasvar.farinosa Schrenk, that is a synonym of P.nivalissubsp.turkestanica, related to *P.longipes* (Lamond 1978). The new taxon differs from P.longipessubsp.longipes by its corolla tube and calyx length ratio and thurm flower characters (Table 1). Differing from other taxa, *P.nivalis*. Subs.turkestanica growing in Kazakhstan, Kirgizstan, Mongolia and Xinjiang, its bracts shape, calyx divided ratio and corolla tube and calyx length of ratio etc. *P.crassifolia*, spread across North Caucasus and Transcaucasus, is similar to the new taxon, but its white-flowered habit is distinctive.

**Table 1.** Morphological comparison of Primulalongipessubsp.bayburtensis with P.longipessubsp.longipes and P.nivalissubsp.turkestanica.

Characters	Primulalongipessubsp.bayburtensis	Primulalongipessubsp.longipes	P.nivalissubsp.turkestanica
Bracts	numerous, linear lanceolate, generally filiform.	several, narrow-triangular.	narrowly lanceolate.
Calyx	tubular, 5–7 mm, divided to 3/5.	campanulate, 6–12 mm, divided to 3/5.	tubular, 6–11 mm, divided to ½.
Calyx teeth	3.5–4.5 mm, lobes linear to lanceolate.	ca. 9 mm, lobes linear to lanceolate.	3–5.5 mm, lobes lanceolate.
Corolla	corolla tube ca. 14 mm, lobes 6–10 mm, ± broadly oblong, entire, magenta.	corolla tube 6–12 mm, lobes 6–9 mm, ± broadly oblong, emarginate or not, lavender-blue to reddish mauve.	corolla tube 8–15 mm, lobes 7–14 mm, oblong, margin entire, violet to purple.
Calyx/Corolla tube length	0.5	0.7	0.4
Thrum flowers:	stamens equalling or above apex of calyx; 1–1.5 mm, style ca. 4 mm.	stamens bearing apex of calyx or above; style ca. 3–3.5 mm.	stamens slightly above apex of calyx; style ca. 2 mm.
Capsule	cylindrical, 2–4.5× as long as calyx.	oblong, 1–2× as long as calyx.	oblong, 1–2× as long as calyx.

It is not surprising to find a new taxon from the province in northeast Türkiye as this area has a rich diversity of *Androsace* and *Primula* species (Lamond 1978; Terzioğlu et. al. 2012; Sefalı 2021; Sefalı and Yapar 2022). P.longipessubsp.bayburtensis is found in Bayburt, Kırklar Mountain, north-eastern Türkiye. The new taxon’s spread is restricted to the southern valley of Kırklar Mountain. This valley is exposed to the Black Sea climate, the air currents from the south and fed by the Çoruh River. Probably, this climate differentiation challenged the new taxon for some adaptation. This environmental factor may be the reason for the new taxon’s changes in the flower futures (calix divided ratio, thurm flowers, corolla tube and calyx length of ratio).

##### Examined specimens.

Primulalongipessubsp.longipes – Türkiye • Gümüşhane, in saxosis tracti Karagolldagh circa lacum Bojuk-goll, 2500 m elev., 31 vii 1894, Sintenis 7307 (E00024014!, L2650049, G, K000732926, BR/BR0000005297337, WU0069730) • Erzurum, Erzurum Province (bordering Rize Province), İspir District (bordering İkizdere District), Kaçkar Dağları (Kaçkar Mountains), about 1.7 km S of Ovit Dağı Geçidi (Ovit-Pass, 2640 m elev.). 40.611027°N, 40.78309°E, C. Gilli (WU11602); Giresun, Karagöl Dağı, 2600 m elev., 10 June 2021, A. Sefalı 690 (VANF 165225) • Rize, Upper Kavrun Valley, Kaçkar Mountains, with *Rhododendroncaucasicum* populations, grazing ground, 2600 m elev., 21 June 2024, A. Sefalı 107? (BIN). P.nivalissubsp.turkestanica – Kyrgyzstan • Talas Oblast, North West of Otmök Pass, 3554 m elev., 12 July 2008, J. Osborne 518 (K000493578). *Primulacrassifolia* – Georgia • (E00024012). Primulalongipessubsp.bayburtensis – Türkiye • Bayburt; Kırklar Mountain, southern valley, river side, stony and wet places, 2950 m elev., 15 June 2024, A. Sefalı 1052 (holotype: VANF!; isotypes: BIN!).

## Supplementary Material

XML Treatment for Primula longipes Freyn & Sint. subsp. bayburtensis

## ﻿Additional information

### Author ORCIDs

Abdurrahman Sefalı https://orcid.org/0000-0002-0092-0857

Yakup Yapar https://orcid.org/0000-0002-5298-0085

İbrahim Demir https://orcid.org/0000-0003-1533-556X

Bayram Yurtvermez https://orcid.org/0000-0001-9726-3672

Ali Murat Keser https://orcid.org/0000-0003-2245-3978

### Data availability

All of the data that support the findings of this study are available in the main text.

## References

[B1] Barrett SC (2019) ‘A most complex marriage arrangement’: recent advances on heterostyly and unresolved questions. New Phytologist 224(3): 1051–1067. 10.1111/nph.16026 31631362

[B2] Darwin C (1877) The Different Forms of Flowers on Plants of the Same Species. John Murray, London UK. 10.5962/bhl.title.46988

[B3] Faegri K Iversen J (1975) Textbook of pollen analysis.

[B4] Fedorov AA (1952) Semeistva pervocvetnye— Primulaceae Vent. Flora SSSR [Flora of the USSR] 18: 108–292.

[B5] Gültepe M Uzuner U Coşkunçelebi K Beldüz AO Terzioğlu S (2010) Internal transcribed spacer (ITS) polymorphism in the wild Primula (Primulaceae) taxa of Türkiye. Turkish Journal of Botany 34(3): 147–157. 10.3906/bot-0905-23

[B6] Hu CM (1994) On the geographical distribution of the Primulaceae. Journal of Tropical and Subtropical Botany 4: 1–14.

[B7] Hu CM Kelso S (1996) Primulaceae. In: Wu CY Raven PH (Eds) Flora of China Volume 15.Science Press, Beijing, and Missouri Botanical Garden Press, St. Louis, 99–185.

[B8] Kato J Inari-Ikeda M Hayashi M Amano J Ohashi H Mii M (2018) *Primula*. Ornamental Crops, 627–647. 10.1007/978-3-319-90698-0_24

[B9] Kelso S (1987) *Primulatschuktschorum* and *Primulaeximia* (Primulaceae: Section Crystallophlomis): a distylous species and its homostylous derivative from the Bering Strait Region, Alaska. Brittonia 63–72. 10.2307/2806976

[B10] Lamond J (1978) *Primula* L. In: Davis PH (Ed.) Flora of Türkiye and the East Aegean Islands Vol.6. Edinburgh University Press, 112–120.

[B11] Menemen Y Kandemir A Aytaç Z (2016) Türkçe Bilimsel Bitki Adları Yönerges. Bağbahçe Bilim Dergisi 3(3): 1–3.

[B12] Morozowska M Czarna A Kujawa M Jagodzinski AM (2011) Seed morphology and endosperm structure of selected species of Primulaceae, Myrsinaceae, and Theophrastaceae and their systematic importance. Plant Systematics and Evolution 291: 159–172. 10.1007/s00606-010-0374-2

[B13] Punt W Hoen PP Blackmore S Nilsson S Le Thomas A (2007) Glossary of pollen and spore terminology. Review of Palaeobotany and Palynology 143(1–2): 1–81. 10.1016/j.revpalbo.2006.06.008

[B14] Rankin DW (2012) 726. *Primulamelanantha*. Curtis’s Botanical Magazine 29(1): 18–33. 10.1111/j.1467-8748.2012.01768.x

[B15] Richards J (2002) *Primula*. New edition. BT Batsford, London.

[B16] Richards J (2003) *Primula*. New edition. In: Sennikov AN (Ed.) 2017. Flora of Uzbekistan, vol. 2. Navro’z Published, Toshkent, 200.

[B17] Sefalı A (2021) *Androsaceazizsancarii* sp. nov. (Primulaceae): a new species from northeastern Anatolia, Türkiye. Nordic Journal of Botany 39(7): 1–8. 10.1111/njb.03208

[B18] Sefalı A Yapar Y (2022) *Androsaceartvinensis* sp. nov. (Primulaceae): A new species from northeastern Anatolia, Türkiye. Phytotaxa 552(3): 171–181. 10.11646/phytotaxa.552.3.1

[B19] Smith WW Fletcher HR (1942) XVII.—The Genus Primula: Section Nivales. Earth and Environmental Science Transactions of The Royal Society of Edinburgh 60(2): 563–627. 10.1017/S0080456800017993

[B20] Terzioğlu S Çoşkunçelebi K Gültepe M (2012) *Primula×uzungolensis* (Primulaceae): a new natural hybrid from NE Anatolia. Turkish Journal of Botany 36(1): 9–19. 10.3906/bot-1011-19

[B21] Thiers B (2025) Index Herbariorum: A global directory of public herbaria and associated staff. New York Botanical Garden’s Virtual Herbarium. http://sweetgum.nybg.org [Retrieved 22 April 2025]

[B22] Wang XJ Barrett SC Zhong L Wu ZK Li DZ Wang H Zhou W (2021) The genomic selfing syndrome accompanies the evolutionary breakdown of heterostyly. Molecular Biology and Evolution 38(1): 168–180. 10.1093/molbev/msaa199 32761213 PMC7782863

[B23] Wu ZK Guo YJ Zhang T Burgess KS Zhou W (2023) *Primulaluquanensis* sp. nov.(Primulaceae), a new species from southwestern China, reveals a novel floral form in the heterostyly-prevailing genus. Plants 12(3): 534. 10.3390/plants12030534 36771618 PMC9918951

